# Comparative analysis of gene ontology-based semantic similarity measurements for the application of identifying essential proteins

**DOI:** 10.1371/journal.pone.0284274

**Published:** 2023-04-21

**Authors:** Xiaoli Xue, Wei Zhang, Anjing Fan

**Affiliations:** 1 School of Science, East China Jiaotong University, Nanchang, China; 2 School of Computer and Information Engineering, Anyang Normal University, Anyang, China; University of Maryland Baltimore, UNITED STATES

## Abstract

Identifying key proteins from protein-protein interaction (PPI) networks is one of the most fundamental and important tasks for computational biologists. However, the protein interactions obtained by high-throughput technology are characterized by a high false positive rate, which severely hinders the prediction accuracy of the current computational methods. In this paper, we propose a novel strategy to identify key proteins by constructing reliable PPI networks. Five Gene Ontology (GO)-based semantic similarity measurements (Jiang, Lin, Rel, Resnik, and Wang) are used to calculate the confidence scores for protein pairs under three annotation terms (Molecular function (MF), Biological process (BP), and Cellular component (CC)). The protein pairs with low similarity values are assumed to be low-confidence links, and the refined PPI networks are constructed by filtering the low-confidence links. Six topology-based centrality methods (the BC, DC, EC, NC, SC, and aveNC) are applied to test the performance of the measurements under the original network and refined network. We systematically compare the performance of the five semantic similarity metrics with the three GO annotation terms on four benchmark datasets, and the simulation results show that the performance of these centrality methods under refined PPI networks is relatively better than that under the original networks. Resnik with a BP annotation term performs best among all five metrics with the three annotation terms. These findings suggest the importance of semantic similarity metrics in measuring the reliability of the links between proteins and highlight the Resnik metric with the BP annotation term as a favourable choice.

## Introduction

Proteins are crucial components of cell and tissue structures and are cornerstones used by an organism to maintain normal life activities. Due to the different roles each protein plays in the life activities of organisms, proteins are divided into essential proteins and nonessential proteins. The deletion or elimination of essential proteins may result in normal cellular function disorders or diseases and may even affect the development and survival of organisms [1, 2]. Previous studies have shown that when a virus attacks the human body, it attacks essential proteins first [3]. For instance, when studying the novel coronavirus, the most important aspect is to determine several possible target proteins and then use super-large computer-aided drug screening to find effective antiviral drugs. Therefore, identifying key proteins has vital application prospects in disease diagnosis [4], drug discovery [5], and drug design [6].

Traditional biological experiments can only be carried out in a limited number of species and are expensive and time consuming [7]. Fortunately, with the rapid development of high-throughput technology, many PPI data have been accumulated, and these provide a convenient condition for identifying essential proteins with computational methods.

PPI networks provide a comprehensive view of the global interaction structure of an organism’s proteome. Initially, the key proteins were predicted by measuring topologic properties. In 2001, Jeong [8] pointed out that proteins involved in more interactions in PPI networks have higher possibilities of being key proteins; this is known as the centrality-lethality rule. Subsequently, a series of topological structure-based approaches were developed, such as the betweenness centrality (BC) [9], eigenvector centrality (EC) [10], neighborhood centrality (NC) [11], subgraph centrality (SC) [12], strength centrality (StrC) [13], average neighbor centrality (aveNC) [14], closeness centrality (CC) [15], information centrality (IC) [16], local average connectivity (LAC) [17], local interaction density (LID) [18], maximum neighborhood component (MNC), density of maximum neighborhood component (DMNC) [19], TP and TP-NC [20]. The performance of these centrality methods depends on the quality of the utilized PPI networks.

PPI networks retrieved from high-throughput techniques are incomplete and inherently noisy [21]. The reliability of yeast two-hybrid assays is approximately 50%, even for the well-studied Saccharomyces cerevisiae species; this impairs the prediction performance of the available topology-based methods.

To overcome the influence of false positive data in PPI networks, two categories of methods have been developed to improve the performance of identifying essential proteins. The first category identifies essential proteins by combining the topological properties of PPI networks with various biological data, such as Gene Ontology (GO) annotation data [22–27], gene expression profiles [23, 25, 27–31], subcellular localizations [24, 25, 31], the domain features of proteins [32], orthologous information [30, 33], and protein complex information [34, 35]. Previous studies have demonstrated that the efficient and effective integration of multiple sources of data could yield better results for identifying essential proteins. For example, Kim [22] proposed that adding gene-level annotation information, such as GO terms, to detect essential proteins would result in higher accuracy than that of existing methods. Li et al. [29] introduced a novel essential protein prediction algorithm named CPPK. CPPK predicts key proteins with a combination of network topology properties and gene expression data. Zhang et al. [23] developed a new method named TEO that combines PPI networks, and gene expression profiles with GO annotation terms, and it achieved higher accuracy in predicting key proteins than previously developed. Peng et al. [32] developed a method called UDoNC that utilize protein domain information and the topology of given PPI network. Lei et al. [24] introduced a novel strategy named RSG using RNA-Seq, GO information, and subcellular localization. Zhang et al. [25] developed TEGS, a new strategy to predict key proteins, which improved prediction accuracy by integrating network topology with subcellular localization information, gene expression profiles, and GO annotation datasets. Peng et al. [33] developed a novel measure to predict key proteins by adding orthologous data. Zhang et al. [30] designed OGN by using gene expressions, orthologies, and network topologies to identify key proteins. Li et al. [34] introduced a novel idea that combines protein complexes information with the topological properties of PPI networks.

The methods in the second category predict key proteins based on refined networks by filtering the false positive interactions in the original network. For instance, Kim et al. [26] designed a motif-based method named MCGO, which utilizes Gene Ontology annotation data to prune several uninformative edges from the given network. Li et al. [31] proposed a novel approach to reconstruct PPI networks by using gene expression information and subcellular localization information. Liu et al. [36] developed a new algorithm, EPPSO, to identify key proteins according to improved particle swarm optimization and reconstructed PPI networks by combining the topology information of the PPI networks with other biological information. Lei et al. [27] presented RWEP, which utilizes GO terms and gene expression data to construct a new weighted PPI network, and a random walk with the restart algorithm is applied to quantify the essentiality value of the protein. Simulation results show that RWEP dominates topology-based approaches in predicting key proteins. However, the performance of these approaches is still unsatisfactory, and many methods are complicated and involve many steps, which might hinder their wide application in biological research.

GO annotation is a system of uniform and normative descriptions of the genes and gene products of all species. A GO annotation collects information on the molecular function (MF), biological process (BP), and cellular component (CC) of different organisms. The GO-based semantic similarity metric (SSM) is a numerical measure that is used to estimate the semantic intimacy between two terms and is widely used for measuring the functional similarities between proteins [37–39]. Five widely used SSMs, Jiang [40], Lin [41], Rel [42], Resnik [43], and Wang [44], are applied to calculate the GO semantic similarity values at present. However, each of the SSMs focuses on characterizing particular aspects of GO annotation terms and has strengths as well as weaknesses. The advantages and disadvantages of these SSMs in evaluating GO semantic similarities are important for predicting key proteins.

In this paper, we comprehensively discuss the aforementioned five semantic similarity measurements in combination with three subontology (BP, CC, and MF) terms on the identification of essential proteins. Six centrality methods (the BC, DC, EC, NC, SC, and aveNC) are applied on refined GO-PPI networks and the results are compared with those of the same methods on the original PPI networks. Extensive comparisons have been conducted under different conditions, and the simulation results offer a reference to biologists when investigating the essential proteins of PPI networks.

## Methods

In this part, six conventional centrality methods (the BC, DC, EC, NC, SC, and aveNC) are reviewed briefly. Then, refined PPI network construction methods are described in detail. Additionally, the utilized datasets and evaluation metrics are presented.

### Centrality methods

PPI networks are abstracted into graph structures, which are denoted as *PPI* = (*P*, *E*), where *P* is composed of proteins and *E* represents the set of interactions between proteins. PPI networks are stored as adjacent matrices. The six centrality calculation methods are calculated as follows.

1. BC
  $$\begin{array}{c} BC\left(p\right)=\sum_{i}\;\sum_{j}\frac{S_{p}\left(i,j\right)}{S(i,j)} \end{array}$$ (1)
  where *S*_*p*_(*i*, *j*) represents the number of shortest paths between protein *i* and *j* that go through protein *p* and *S*(*i*, *j*) represents the number of shortest paths between protein *i* and protein *j*. Considering the global characteristics of PPI networks, this method can identify some nodes whose degrees are not high but play a vital role in the connection of the given network.
2. DC
  $$\begin{array}{c} DC\left(p\right)=\text{deg}\left(p\right)=\sum_{u}a_{p,u} \end{array}$$ (2)
  where deg(*p*) represents the number of proteins connected to *p* directly, which is called the degree of *p*. And *a*_*p*,*u*_ ∈ *A* represents the interactions between proteins *p* and *u*.
3. EC
  $$\begin{array}{c} EC\left(p\right)=\alpha_{\text{max}}\left(p\right) \end{array}$$ (3)
  where *α* is a eigenvector of the adjacency matrix *A* and *α*_max_(*p*) is the *p*th component of the eigenvector belonging to the maximum eigenvalue λ_max_.
4. NC
  $$\begin{array}{c} NC\left(p\right)=\sum_{u\in N_{p}}ECC_{pu}=\sum_{u\in N_{p}}\frac{|N_{p}⋂N_{u}|}{\text{min}(|N_{p}|-1,|N_{u}|-1)} \end{array}$$ (4)
  where *N*_*p*_ and *N*_*u*_ represent the neighboring sets of proteins *p* and *u*, respectively. ECC is the edge clustering coefficient. This method characterizes the connection relationships between a node and its neighbors; that is, the similarity of the relationship between two proteins is described by calculating the number of common neighbor nodes.
5. SC
  $$\begin{array}{c} SC\left(p\right)=\sum_{l=0}^{\infty }\frac{\mu_{l}\left(p\right)}{l!} \end{array}$$ (5)
  where *μ*_*l*_(*p*) represents the number of loops whose starting and ending proteins are *p* and the lengths of these loops are l. In complex networks, essential proteins tend to form dense subgraphs. The shorter the loop is, the more likely the protein is to be in a dense subgraph and to be a key protein.
6. aveNC
  $$\begin{array}{c} aveNC\left(p\right)=\frac{\sum_{u\in N_{p}}\text{deg}(u)}{\text{deg}(p)} \end{array}$$ (6)
  where *N*_*p*_ represents the set of protein *p*’s neighbors. The significance of a protein is measured by its neighbors.

### Constructing refined PPI network by applying GO-based SSMs

There are two kinds of measures used to record confidence scores for a PPI network. One relies on interaction data [45], and the other takes gene expression values [46], functional similarities [39, 47], and other information into consideration [37]. According to the basic idea that proteins interacting in the same cell have a higher possibility of being involved in a similar biological process than that do not interact, we assume that the protein pairs with smaller semantic similarity values are more likely to be false positive links.

Five widely used methods, Jiang, Lin, Rel, Resnik, and Wang, are applied to compute semantic similarities based on the GO terms between proteins, and these are denoted as confidence scores. Wang determines the confidence scores between two proteins according to the locations of their corresponding GO terms in the GO graph and their ancestor terms’ relationships. The other four methods are based on information content (IC), which depends on the probabilities of the two GO terms involved and their closest common ancestor terms in the corpus of the GO annotation information.

The details of the five SSMs (semantic similarity metric) are shown as follows:

1. Resnik
  Resnik believes that information content (IC) is the most informative common ancestor (MICA) [48]. The similarity between protein pairs *m* and *v* in this method is denoted as
  $$\begin{array}{c} SSM_{Resnik}\left(m,v\right)=\underset{t\in C}{\text{max}}\;IC\left(t\right)=IC\left(MICA\left(m,v\right)\right) \end{array}$$ (7)
  where *C* represents the set of common ancestors of *m* and *v*. The IC mentioned above is denoted as *IC*(*t*) = −ln*p*(*t*), where *p*(*t*) represents the probability of occurrence in the GO corpus and IC is used to express the specificity of a protein.
2. Lin and Jiang
  It seems that the performance of Resnik is valid for calculating the similarity of two terms, but it cannot distinguish between terms that have the same MICA. To tackle this problem, Lin and Jiang developed new methods with comprehensive consideration of the ICs between protein pairs and their MICAs. The similarity of two proteins based on the Lin and Jiang methods is defined as
  $$\begin{array}{c} SSM_{Lin}\left(m,v\right)=\frac{2\times IC(MICA(m,v))}{IC(m)+IC(v)} \end{array}$$ (8)
  $$\begin{array}{c} SSM_{Jiang}\left(m,v\right)=1-\left[IC\left(m\right)+IC\left(v\right)-2\times IC\left(MICA\left(m,v\right)\right)\right] \end{array}$$ (9)
3. Rel
  Shortcomings still exist in the approaches developed by Lin and Jiang. The similarity between two terms is overestimated when a protein is an ancestor of another. In addition, these approaches ignore the specificities of the two terms. By combining Resnik and Lin, Rel presented a novel measure to capture the similarity between two terms. The similarity between two proteins is defined as
  $$\begin{array}{c} SSM_{Rel}\left(m,v\right)=\frac{2\times IC(MICA(m,v))(1-p(MICA(m,v)))}{IC(m)+IC(v)} \end{array}$$ (10)
4. Wang
  Wang is a hybrid method that combines the number of common ancestors with the locations of these ancestors in the GO graph when calculating the similarity between two terms. GO terms are presented as directed acyclic graphs (DAGs). Suppose that *G*_*v*_ = (*P*_*v*_, *E*_*v*_) is a GAG for a GO term *v*, where *P*_*v*_ contains the ancestor terms of *v* including itself, and *E*_*v*_ is composed of edges that connect the GO terms in *G*_*v*_. Other terms closer to *v* in *G*_*v*_ contribute more to its semantics. The contribution of a protein *u* to the semantics of protein *v* in *G*_*v*_ is defined as the S-value of *u* and is calculated as
  $$\begin{array}{c} \left\{ \begin{array}{c} S_{G_{v}}(v)=1\\ S_{G_{v}}(u)=\text{max}\left\{w_{e}\times S_{G_{v}}\left(u^{\prime }\right)|u^{\prime }\in child\;of\;u\right\}\;\;if\;u\neq v \end{array}\right. \end{array}$$ (11)
  where *ω*_*e*_(0 < *ω*_*e*_ < 1) is the semantic contribution factor for edge *e* ∈ *E*_*v*_ that links term *u* with its child term *u*′. And *SV*(*v*) is used to compare the semantics of two GO terms, and *SV*(*v*)is defined as
  $$\begin{array}{c} SV\left(v\right)=\sum_{u\in P_{v}}S_{G_{v}}\left(u\right) \end{array}$$ (12)

The semantic similarity between protein pairs *m* and *v* is denoted as

$$\begin{array}{c} SSM_{Wang}\left(m,v\right)=\frac{\sum_{u\in P_{m}⋂P_{v}}\left(S_{G_{m}}\left(u\right)+S_{G_{v}}\left(u\right)\right)}{SV(m)+SV(v)} \end{array}$$ (13)

**Fig 1:**
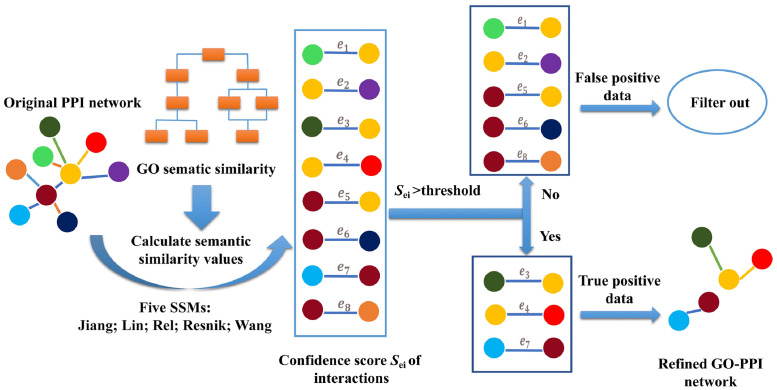
The process of constructing a GO-PPI network.

In this article, we apply five GO-based semantic similarity measurements to measure the reliability of protein pairs. For each SSM, we first compute the confidence scores for all of the protein pairs, and then construct refined PPI networks by filtering the interactions with low confidence scores. The refined PPI networks we obtain by measuring the GO semantic similarity are named GO-PPI for short, and the network refined by using the Resnik metric under the BP annotation term is named Resnik-BP GO-PPI for short. The main idea of constructing a refined GO-PPI network is shown in Fig 1.

### Experimental data

To compare the performance of these centrality methods under different combinations of strategies, we choose the well-studied Saccharomyces cerevisiae PPI data for experiments, as they are widely applied for testing the performance of new methods. The datasets include the YDIP dataset composed of 5093 proteins and 24743 interactions, the new DIP dataset, which includes 4928 proteins and 17201 interactions, the Krogan dataset containing 7123 interactions among 2708 proteins, and the Krogan Extended dataset, which consists of 3672 proteins with 14317 interactions. A summary of these datasets is presented in Table 1.

**Table 1:** The detailed information of four PPI datasets.

Dataset	Proteins	Interactions	Essential proteins	Density
YDIP	5093	24743	1167	0.0019
DIP PPI	4928	17201	1150	0.0014
Krogan	2708	7123	786	0.0019
Krogan Extended	3672	14317	929	0.0021

The GO annotation information of each protein is downloaded from the Saccharomyces Genome Database, which was released on September 10th, 2020.

The benchmark of a known essential protein dataset including 1285 proteins is collected from four different databases (MIPS [49], SGD [50], DEG [51], and SGDP (http://www.sequence.stanford.edu/group/).

### Evaluation metrics

To measure the efficiency of the proposed strategy, we calculate the numbers of key proteins predicted correctly among the top 600 ranked proteins, and the corresponding prediction precisions of the six topology-based methods are also calculated under the original PPI network and refined GO-PPI network. The prediction precision is denoted as

$$\begin{array}{c} \text{Pr}ecision=\frac{TP}{TP+FP} \end{array}$$ (14)

where *TP* describes the number of true positives, and *FP* describes the number of false positives.

## Results and discussion

To evaluate whether the performance of the reconstructed GO-PPI network is better than that of the corresponding original PPI network in identifying key proteins, six topology structure-based methods (the BC, DC, EC, NC, SC, and aveNC) are applied in the experiments. We compare the numbers of key proteins identified properly and the prediction precisions under different types of strategies. The threshold for GO semantic similarity is set to 0.33 for filtering the unreliable links in the PPI networks.

### Analysis of the original network and refined GO-PPI network

The number of interactions in a network influences the speed of calculation for identifying essential proteins. The lower the number of interactions, the less time is required for the calculation. Therefore, we compute the number of interactions and the portions of key proteins under the original PPI network and refined the GO-PPI network for the YDIP dataset. As shown in Table 2, the number of interactions declines dramatically after filtering the links with low-confidence scores, and more than half of the interactions are filtered, so the computational efficiency is greatly improved. Furthermore, the numbers of proteins and key proteins are reduced, but the portion of essential proteins is increased, which is more beneficial for identifying key proteins. For example, in networks with the application of the Resnik metric, the proportions of essential proteins under the three subontologies (the BP, CC, and MF) reach 39.83%, 41.55%, and 37.82%, respectively, while they are 22.91% in the original PPI network.

**Table 2:** The number of interactions and the portions of essential proteins under the original PPI network and GO-PPI network for the YDIP dataset.

Ontology	SSMs	Network	No. interactions		No. proteins		Portion of essential proteins
			Reserved	Filtered	Nonessential	Essential	
		original PPI	24743	0	5093	1167	22.91%
BP	Jiang	GO-PPI	5456	19287	2313	785	33.94%
	Lin	GO-PPI	6323	18420	2522	833	33.03%
	Rel	GO-PPI	5746	18997	2428	825	33.98%
	Resnik	GO-PPI	3336	21407	1725	687	**39.83%**
	Wang	GO-PPI	5418	19325	2644	864	32.68%
CC	Jiang	GO-PPI	11762	12981	3535	951	26.90%
	Lin	GO-PPI	8408	16335	3107	900	28.97%
	Rel	GO-PPI	6140	18603	2760	849	30.76%
	Resnik	GO-PPI	2018	22725	1160	482	**41.55%**
	Wang	GO-PPI	18082	6661	4182	1069	25.56%
MF	Jiang	GO-PPI	3640	21103	1821	615	33.77%
	Lin	GO-PPI	3177	21566	1733	594	34.28%
	Rel	GO-PPI	2821	21922	1667	585	35.09%
	Resnik	GO-PPI	1331	23412	1092	413	**37.82%**
	Wang	GO-PPI	4902	19841	2733	792	28.98%

In the meantime, we study the interactions that rank among the top 600. As the numbers of interactions are different for the original network and the reconstructed GO-PPI network, we compute the proportions of the interactions between essential protein pairs (Ess-ess), essential and nonessential protein pairs (Ess-noness), and nonessential pairs (Noness-noness), and the results for the YDIP dataset under the BP subontology are shown in Table 3. It can be seen that the portion of Ess-ess interactions is significantly improved under the five refined GO-PPI networks, and the portions of Ess-noness and Noness-noness interactions under the GO-PPI network are much lower than those under the original PPI network. We can also see that Wang achieves the best performance compared with those of the other four SSMs. For instance, the portion of essential pairs reaches 57.99% when using the NC method under Wang, which is the highest for the six different networks. And the interactions between the essential and nonessential pairs are only 12.87% of total interactions under Wang versus 37.27% under the original PPI network for the SC method.

**Table 3:** The portions of interactions under the original PPI network and GO-PPI network for the YDIP dataset (BP).

SSMs	Network	Interactions	BC	DC	EC	NC	SC
	original PPI	Ess-ess	17.11%	20.03%	17.21%	32.11%	17.21%
		Ess-noness	37.64%	35.91%	37.27%	28.12%	37.27%
		Noness-noness	45.25%	44.06%	45.52%	39.77%	45.52%
Jiang	GO-PPI	Ess-ess	34.85%	46.72%	47.32%	55.98%	49.46%
		Ess-noness	**28.34%**	20.12%	17.41%	**16.87%**	15.94%
		Noness-noness	36.82%	33.16%	35.27%	27.14%	34.60%
Lin	GO-PPI	Ess-ess	31.87%	45.07%	47.65%	53.64%	48.64%
		Ess-noness	28.85%	20.15%	18.05%	17.38%	17.42%
		Noness-noness	39.28%	34.78%	34.30%	28.98%	33.94%
Rel	GO-PPI	Ess-ess	33.51%	47.71%	52.74%	56.22%	52.50%
		Ess-noness	28.95%	20.18%	15.10%	17.12%	15.59%
		Noness-noness	37.53%	32.12%	32.16%	26.66%	31.91%
Resnik	GO-PPI	Ess-ess	34.85%	46.72%	47.32%	55.98%	49.46%
		Ess-noness	**28.34%**	20.12%	17.41%	**16.87%**	15.94%
		Noness-noness	36.82%	33.16%	35.27%	27.14%	34.60%
Wang	GO-PPI	Ess-ess	**35.57%**	**51.87%**	**55.39%**	**57.99%**	**55.99%**
		Ess-noness	29.18%	**17.90%**	**13.79%**	17.06%	**12.87%**
		Noness-noness	**35.24%**	**30.23%**	**30.81%**	**24.95%**	**31.14%**

### Comparison of the numbers of true predictions under different strategies

In this part, we do a systematic evaluation of the performance of the newly constructed networks on the four test datasets. For each dataset, we adopt five SSMs to calculate confidence scores for the protein pairs in the PPI network under the three GO annotation terms (the BP, MF, and CC) and obtain fifteen kinds of refined GO-PPI networks. Six centrality methods are applied to predict the key proteins of the newly constructed GO-PPI network and the original PPI network.

Table 4 presents the numbers of essential proteins correctly identified from the top 600 candidate proteins of the original network and refined GO-PPI network with different SSMs under the three sub-ontologies (the BP, CC, and MF). As seen from Table 4, for the YDIP dataset, the numbers of essential proteins correctly identified under the six centrality methods on each of the newly constructed GO-PPI networks are consistently larger than those under the corresponding original PPI networks. For example, compared to the original network, the EC method yields an improvement of 57.01% on the Wang-BP (the Wang method under BP subontology) network, and the aveNC method provides an improvement of 300% on the Resnik-BP network. In terms of three subontologies, the performance of these methods under the refined GO-PPI network obtained with BP annotation term is significantly better than it under CC and MF annotation terms, especially for the Resnik and Wang methods.

**Table 4:** The numbers of essential proteins detected by the six centrality methods under different strategies for the YDIP dataset (top 600).

Ontology	SSMs	Network	BC	DC	EC	NC	SC	aveNC
		original PPI	220	251	221	309	221	80
BP	Jiang	GO-PPI	266	327	310	351	324	221
	Lin	GO-PPI	257	329	332	349	334	201
	Rel	GO-PPI	266	336	344	353	341	222
	Resnik	GO-PPI	**311**	**363**	340	351	**349**	**320**
	Wang	GO-PPI	265	344	**347**	**356**	346	259
CC	Jiang	GO-PPI	222	253	217	325	214	100
	Lin	GO-PPI	246	291	237	321	247	124
	Rel	GO-PPI	237	299	263	318	274	173
	Resnik	GO-PPI	**301**	**317**	**280**	315	**291**	**295**
	Wang	GO-PPI	229	269	228	**328**	228	92
MF	Jiang	GO-PPI	**239**	272	246	276	249	184
	Lin	GO-PPI	238	269	240	**291**	249	216
	Rel	GO-PPI	237	267	248	289	**254**	227
	Resnik	GO-PPI	237	261	238	242	243	**234**
	Wang	GO-PPI	230	**278**	**259**	289	232	145

To verify the superiority of the newly proposed strategy, we calculate the number of key proteins identified correctly by each method under three GO subontology terms for the reduced DIP PPI dataset, the Krogan dataset, and the Krogan Extended dataset. The calculation results are listed in Tables 5–7.

**Table 5:** The number of essential proteins detected by the six centrality methods under different strategies for the new DIP dataset (top 600).

Ontology	SSMs	Network	BC	DC	EC	NC	SC	aveNC
		original PPI	239	274	160	318	163	72
BP	Jiang	GO-PPI	285	347	337	351	333	258
	Lin	GO-PPI	275	350	332	346	329	228
	Rel	GO-PPI	282	358	345	349	345	261
	Resnik	GO-PPI	**355**	**370**	**348**	351	345	**340**
	Wang	GO-PPI	285	349	336	**352**	**349**	285
CC	Jiang	GO-PPI	226	289	148	315	201	123
	Lin	GO-PPI	253	309	203	311	293	171
	Rel	GO-PPI	261	**319**	279	303	294	212
	Resnik	GO-PPI	**298**	309	**280**	308	**321**	**301**
	Wang	GO-PPI	252	294	216	**340**	272	94
MF	Jiang	GO-PPI	244	276	261	285	256	226
	Lin	GO-PPI	260	276	254	288	260	230
	Rel	GO-PPI	**265**	**286**	**278**	286	**279**	**241**
	Resnik	GO-PPI	231	250	239	242	248	240
	Wang	GO-PPI	242	282	256	**306**	225	171

**Table 6:** The number of essential proteins detected by the six centrality methods under different strategies for the Krogan dataset (top 600).

Ontology	SSMs	Network	BC	DC	EC	NC	SC	aveNC
		original PPI	227	288	228	305	242	141
BP	Jiang	GO-PPI	302	317	268	325	278	297
	Lin	GO-PPI	298	320	287	324	304	290
	Rel	GO-PPI	305	329	290	323	300	293
	Resnik	GO-PPI	**311**	328	299	312	305	**325**
	Wang	GO-PPI	308	**336**	**309**	**336**	**307**	301
CC	Jiang	GO-PPI	217	279	245	309	255	236
	Lin	GO-PPI	236	290	250	309	281	268
	Rel	GO-PPI	262	295	264	**311**	291	282
	Resnik	GO-PPI	**300**	**309**	**289**	305	**305**	**304**
	Wang	GO-PPI	235	292	243	300	263	170
MF	Jiang	GO-PPI	266	270	254	266	254	269
	Lin	GO-PPI	266	273	247	271	260	269
	Rel	GO-PPI	**271**	**278**	**263**	**273**	**272**	**275**
	Resnik	GO-PPI	252	254	256	254	260	257
	Wang	GO-PPI	264	276	229	264	240	251

**Table 7:** The number of essential proteins detected by the six centrality methods under different strategies for the Krogan Extended dataset (top 600).

Ontology	SSMs	Network	BC	DC	EC	NC	SC	aveNC
		original PPI	240	271	227	305	227	103
BP	Jiang	GO-PPI	264	324	277	326	280	253
	Lin	GO-PPI	265	323	284	328	297	251
	Rel	GO-PPI	264	327	276	**329**	299	266
	Resnik	GO-PPI	**317**	327	306	326	307	**305**
	Wang	GO-PPI	275	**332**	**317**	327	**316**	288
CC	Jiang	GO-PPI	215	259	215	**313**	217	149
	Lin	GO-PPI	219	276	250	304	267	190
	Rel	GO-PPI	231	286	270	305	282	254
	Resnik	GO-PPI	**296**	**301**	**288**	303	**283**	**308**
	Wang	GO-PPI	253	275	242	309	241	147
MF	Jiang	GO-PPI	253	273	249	267	251	229
	Lin	GO-PPI	**266**	280	**263**	250	250	247
	Rel	GO-PPI	265	**285**	258	255	250	254
	Resnik	GO-PPI	258	259	250	251	**254**	**257**
	Wang	GO-PPI	227	281	235	**276**	241	183

For the new DIP PPI dataset, the comparison results are shown in Table 5. We can observe that the six centrality methods perform best under the refined GO-PPI network constructed by using the Resnik metric with the BP subontology, suggesting that this network is relatively more accurate and complete than it is under the MF and CC subontologies.

However, for the MF and CC subontologies, some of the centrality methods perform poorly under the refined GO-PPI network, such as the BC method under the Jiang-CC (the Jiang method under the CC subontology) PPI network and the DC method under the Resnik-MF (the Resnik method under the MF subontology) PPI network. The maximum number of essential proteins predicted by the NC method in all five newly constructed PPI networks under the MF subontology is 306, which is compared to the 318 correctly predicted essential proteins under the original PPI network. Considering the number of interactions under the refined GO-PPI network in the MF subontology (Table 2), this is might due to the GO annotation under MF is incomplete for the protein pairs in the new DIP dataset; therefore, the confidence scores of many true interacting protein pairs are assigned to 0, and the refined network constructed by using the five SSMs is relatively sparse, which hinders the performance of the NC centrality approach in identifying key proteins.

As seen from the results obtained using the Krogan dataset in Table 6, the performance of these six centrality methods under the refined GO-PPI networks constructed by using the five SSMs with the BP and CC annotation terms dominates the number of true key proteins predicted under the original networks. In particular, under the GO-PPI network filtered by the Wang method under the BP term, the numbers of correctly identified proteins achieved by the two centrality methods (the DC and NC) reach 336, which is significantly larger than that on the original PPI network. For the CC annotation term, the network filtered by using the Resnik metric is relatively more precise than other methods in predicting key proteins. Compared to the number of correct predictions obtained under the original PPI network, more than half of the centrality methods performed better under the newly constructed network with the MF sub-annotation term, except for the DC and NC methods.

Similar results are obtained on the Krogan Extended dataset and listed in Table 7. The number of key proteins truly predicted under the newly refined GO-PPI networks constructed with the BP subontology is consistently larger than that under the original PPI networks, and the refined network dominates the the network constructed with the CC and MF subontologies in terms of performance.

**Fig 2:**
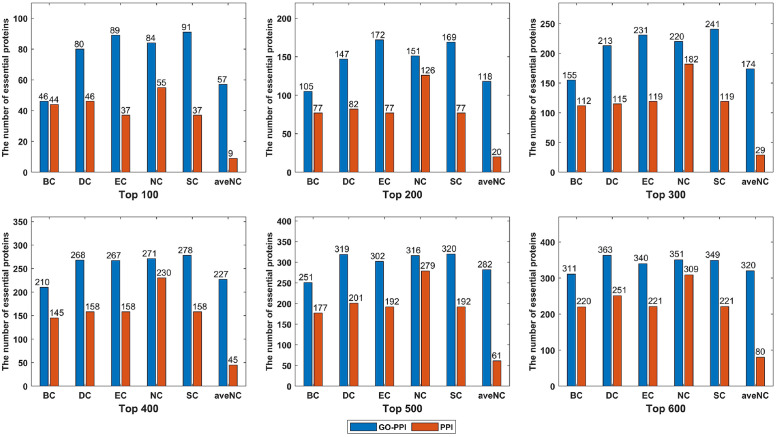
The numbers of key proteins predicted correctly under the original PPI network and reconstructed GO-PPI network for the YDIP dataset.

To further investigate the performance of the six centrality methods under the newly refined networks, we take the network constructed by using the Resnik metric with BP subontology for the YDIP dataset as an example. We calculate the numbers of key proteins predicted by these centrality approaches among the top 100, 200, 300, 400, 500, and 600 ranked candidates. As shown in Fig 2, the performance of these six topology-based methods is highly improved under the reconstructed GO-PPI network in terms of the number of key proteins identified correctly. Particularly, for the SC method, 91 out of 100 candidate predicted proteins are correctly identified, which is significantly more than those predicted by all of the other state-of-the-art approaches. When compared to the results of the original PPI network, 85.22% and 94.12% improvements are still achieved by the DC and EC methods under the GO-PPI networks for the top 300 candidates. For the SC and aveNC approaches, the improvements yielded are both greater than 100% with the application of the GO-PPI networks when predicting the top 300 candidate proteins.

### Comparison of prediction precision for the six centrality methods

To validate the advantage of the reconstructed GO-PPI network in predicting key proteins intuitively, six centrality approaches (the BC, DC, EC, NC, SC, and aveNC) are taken to predict key proteins under the original PPI network and reconstructed GO-PPI network.

**Fig 3:**
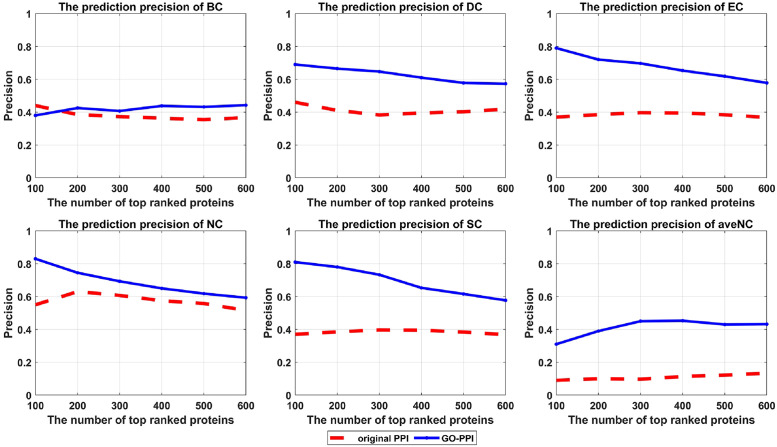
Prediction precision of six centrality methods under the original PPI network and reconstructed GO-PPI network for the YDIP dataset.

Fig 3 shows the prediction precision comparison for the six centrality approaches under the original PPI network and GO-PPI network reconstructed by using the Wang method with the BP subontology information for the YDIP dataset. Fig 3 shows that the prediction precisions of these six methods under the newly constructed GO-PPI network show significant improvements over those obtained with the original PPI network.

### Comparison of ROC curves

**Fig 4:**
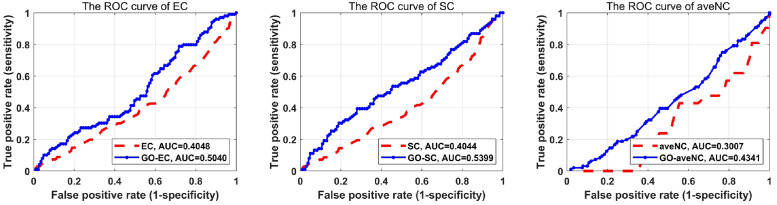
The comparison of ROC curves for original new DIP PPI network and Wang-BP (the Wang method under the BP subontology) PPI network under (left) EC method, (middle) SC method and (right) aveNC method.

**Fig 5:**
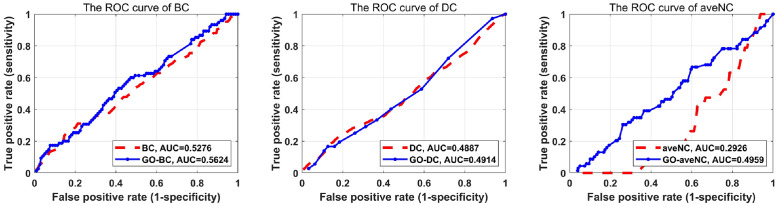
The comparison of ROC curves for original YDIP PPI network and Resink-BP (the Resink method under the BP subontology) PPI network under (left) BC method, (middle) DC method and (right) aveNC method.

To further exhibit the performance of proposed strategy, we compared the ROC curves of different methods under original PPI networks and corresponding GO-PPI networks. The top 600 ranked proteins predicted by each method are assumed as essential, the rest proteins are non-essential. For the gold-standard essential proteins in GO-PPI is obtained from the original true essential protein sets and filtered the proteins that are not in GO-PPI network. The rank value of each protein in original PPI network and GO-PPI network are normalized, and the true positive rate as well as false positive rate is calculated by using the threshold value varies in [0, 1]. We draw the ROC curve by using the obtained true positive rate and false positive fate. AUC means the area under the ROC curve and calculated by using trapz function in Matlab. The comparison of ROC curves as well as AUC value under original new DIP PPI and YDIP PPI network are shown as following Figs 4 and 5. As shown in Figs 4 and 5, the ROC curves under GO-PPI network is higher than the corresponding original PPI network, suggesting that the GO-PPI network we constructed is reliable for predicting essential proteins.

### Analysis of the effect of the threshold

Since the new GO-PPI network is constructed by filtering the unreliable links in the original PPI network, we need to choose an appropriate threshold to distinguish false positive data and real interactions. However, the threshold value is related to the SSMs and the quality of a given PPI network, and different thresholds should be set for different SSMs to achieve the best performance.

**Fig 6:**
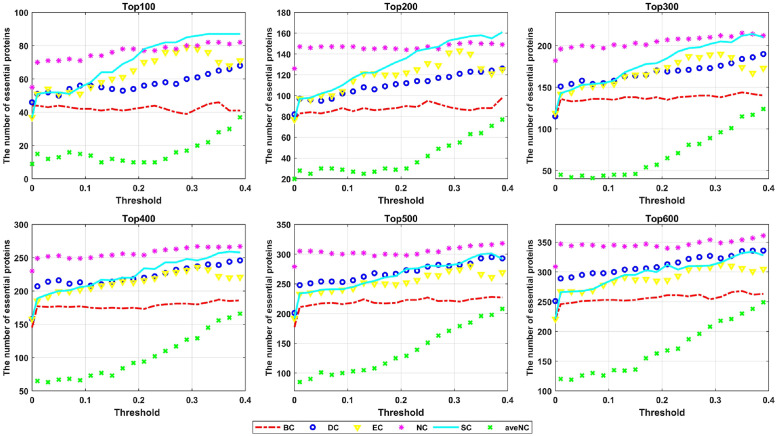
The numbers of correctly identified key proteins among the top 100, 200, 300, 400, 500, and 600 candidates for different method vs the threshold value.

To investigate the effect of the threshold on the performance of the methods in identifying essential proteins, we plot the true numbers of key proteins identified among the top 100, 200, 300, 400, 500, and 600 candidates as functions of the threshold value for the YDIP Jiang-BP network in Fig 6. As shown in Fig 6, the numbers of correct predictions increase with increasing threshold value for all of the methods, especially the DC, SC, and aveNC methods. The results show that GO semantic similarity is efficient in filtering unreliable links in PPI networks, and almost all of the considered methods achieve the maximum number of correctly predicted essential proteins with a relatively large threshold value.

## Conclusions

Predicting essential proteins by developing computational methods from PPI networks has been a hot topic in recent years. However, the PPIs obtained by high-throughput technology at present have high false positive rates. False interactions in PPI networks have great effects on the performance of computational methods in terms of predicting key proteins. Semantic similarity measures have been shown to be useful for assessing the confidence scores between linked protein pairs. The best of the five current widely used semantic similarity measurements for selecting appropriate metrics to measure the reliability of interactions remains unclear.

This paper presents a comparison between GO-PPI networks newly constructed by five semantic similarity methods with three GO annotation terms and corresponding original PPI networks. The six topological-based centrality methods (the BC, DC, EC, NC, SC, and aveNC) are used to calculate the numbers of correct predictions and the precisions for the 600 top-ranked candidate proteins under the newly constructed GO-PPI networks and original networks. The comparison results suggest that the prediction accuracies under each of the newly constructed GO-PPI networks are consistently higher than those under the original PPI network. In particular, the networks constructed by using the semantic similarity metrics of Resnik and Wang under the BP annotation term are most reliable for predicting essential proteins among these topological-based centrality methods. These results suggest that constructing a new PPI network by using the Resnik and Wang metrics under the BP annotation term can filter out some false positive data effectively and improve the quality of the network, which is also the direction of future research.
